# Clinic evaluation of cognitive impairment in post-COVID syndrome: Performance on legacy pen-and-paper and new digital cognitive tests

**DOI:** 10.1016/j.bbih.2024.100917

**Published:** 2024-12-01

**Authors:** Aysha Mohamed Rafik Patel, Gina Gilpin, Anna Koniotes, Catherine Warren, Cian Xu, Paul W. Burgess, Dennis Chan

**Affiliations:** a Institute of Cognitive Neuroscience, University College London, London, United Kingdom; b Department of Neurology, University Hospitals Sussex NHS Trust, United Kingdom

**Keywords:** Post-COVID syndrome, Cognitive impairment, Brain fog, Addenbrooke's cognitive examination III

## Abstract

**Background:**

Cognitive impairment, colloquially termed “brain fog”, is one of the most prevalent manifestations of post-Covid syndrome and a major contributor to impaired daily function and reduced quality of life. However, despite the high numbers of affected individuals presenting to clinical services with cognitive impairment, little work has been undertaken to date on the suitability of current memory clinic tests for identifying the cognitive deficits in this new acquired cognitive disorder.

The aim of this study was therefore to determine the performance of people with post-Covid syndrome presenting with cognitive impairment on the Addenbrooke's Cognitive Examination-III (ACE-III), a cognitive test used widely in memory clinics. A subset of individuals also underwent testing on a novel battery of short digital tests assessing attention, speed of information processing and executive function, representing the domains primarily implicated in post-Covid cognitive dysfunction.

**Methods:**

102 individuals with post-Covid syndrome presenting with subjective cognitive complaints were recruited from a specialist cognitive long Covid clinic at University Hospitals Sussex NHS Trust. All participants completed self-report questionnaires on depression, anxiety, sleep and fatigue. Cognitive performance was assessed using the ACE-III, with 20 participants also being tested on the digital Long COVID Assessment Battery (LCCAB) (*N* = 20).

**Results:**

The overall sample had a mean ACE-III score of 91/100 (SD 6) with 15.7% (16/102) scoring at or below the cut-off score considered to represent objective cognitive impairment. Of the 20 individuals who also completed the LCCAB, 89.47% were impaired on at least one task, primarily in the domains of attention, executive function and processing speed. Cognitive performance was not associated with depression, anxiety, sleep quality or fatigue.

**Conclusion:**

The vast majority of individuals with post-Covid syndrome presenting with subjective cognitive complaints do not exhibit impaired performance on the ACE-III. This likely reflects the historical use of ACE-III and other pen and paper cognitive tests to detect cognitive impairment in diseases causing dementia, but they are ill-equipped to identify impairment in those cognitive domains affected in post-Covid syndrome. The LCCAB detected cognitive impairments in nearly 90% of participants, primarily affecting attention, executive function, and processing speed. These observations highlight the need for alternative cognitive tests for use in routine clinical practice to detect the impairments in new acquired cognitive disorders that are not adequately captured by legacy tests.

## Highlights

• Post-Covid cognitive impairment affects daily function and quality of life.
• ACE-III fails to detect cognitive impairment in most post-Covid patients.
• 89% of patients have impaired attention, executive function, processing speed on novel tests.
• New tests are needed for post-Covid cognitive assessment in clinical practice.

## 1 Introduction

Globally, the World Health Organisation (WHO) has reported to date over 772 million laboratory-confirmed COVID-19 cases, of which a conservative estimate of 10% struggle with post-Covid syndrome, previously termed “long COVID”, in which symptoms persist over three months after the acute infection (Davis et al., 2023). “Brain fog” represents one of the commonest reported symptoms with those affected describing forgetfulness, slower processing, disorientation, difficulties in multitasking and word-finding (Callan et al., 2022; Nalbandian et al., 2021; Ziauddeen et al., 2022). This cognitive impairment not only diminishes quality of life but also affects independence, work and psychosocial functioning (Davis et al., 2021; Graham et al., 2021; Varatharaj et al., 2020), with major negative consequences both for affected individuals and the wider socioeconomic landscape.

Cognitive function in post-Covid syndrome has since been assessed within large-scale studies (Hampshire et al., 2024). Application of in-person and web-based neuropsychological batteries has shown executive function (Braga et al., 2022; Delgado-Alonso et al., 2022; Hampshire et al., 2024; Henneghan et al., 2022), attention (Ferrucci et al., 2021, 2022; Graham et al., 2021; Guo et al., 2022; Zhao et al., 2022; Zhou et al., 2020), speed of information processing (Becker et al., 2021; Ferrucci et al., 2022; Zhao et al., 2024) and episodic memory (Delgado-Alonso et al., 2022; Serrano-Castro et al., 2022; Zhao et al., 2022) to be the most severely and consistently affected domains. However, such comprehensive cognitive testing is ill-suited to routine clinical practice in view of high demands in terms of test duration, availability of test material and test burden, the latter being of particular relevance given the high prevalence of post-exertional fatigue in post-Covid syndrome. Instead, when patients present with cognitive symptoms, the tests routinely available in clinic are the Montreal Cognitive Assessment (MoCA; (Nasreddine et al., 2005), Mini Mental Status Examination (MMSE (Folstein et al., 1975); and the Addenbrooke's Cognitive Examination III (ACE-III; (Hsieh et al., 2013). These tests, designed primarily for assessing Alzheimer's disease and other dementia-causing conditions, focus predominantly on cognitive domains affected in these disorders: episodic memory, spatiotemporal orientation, language, and higher visual processing. The suitability of such tests for assessing cognitive function in post-Covid syndrome is less clear. Attention, processing speed and executive function are the domains most frequently and most severely affected in people presenting with post-Covid syndrome, but are either assessed only in part (attention, executive function) or not at all (processing speed) within the MoCA, MMSE or ACE-III. Those studies to date that have applied such tests to this patient cohort have only been able to report global cognitive scores rather than a detailed cognitive profile (Ceban et al., 2022; Crivelli et al., 2022), with the frequency of cognitive impairment identified with these tests varying widely, ranging from no measurable impairment (Mattioli et al., 2021; Petersen et al., 2022) to 80% (Alemanno et al., 2021). While this variability could be attributed to differences in sample characteristics, such as study size and assessment times, more recent studies suggest that the MMSE and MoCA cannot reliably detect cognitive dysfunction in this patient cohort (Mattioli et al., 2021; Petersen et al., 2022; Schild et al., 2023). For instance, in one study (N = 52), global mean cognitive scores did not indicate group-level impairment (MMSE: 29.7, cut-off <27; MoCA: 27.3, cut-off ≤ 25), and only a limited number of participants exhibited objective cognitive impairment on the MMSE (*n* = 1*)* and MoCA (*n* = 13) despite self-reported cognitive complaints. In contrast, when the same participants underwent comprehensive neuropsychological testing, 59.6% (*n* = 31) displayed objective cognitive deficits (Schild et al., 2023). Similarly, multiple studies administering the ACE-III at various time-points post COVID-19 infection have reported no group-level impairment, with global mean cognitive scores ranging between 87.27 and 92 (Charoenporn et al., 2024; Kausel et al., 2024; Omar et al., 2022).

As a consequence, there is an unmet need for tests that not only reflect the cognitive symptoms experienced in post-Covid syndrome but are also applicable within a routine clinic setting given the time and resource limitations that preclude use of full neuropsychological test batteries. The requirement for brief tests with low user burden is amplified by the need to mitigate against the post-exertional fatigue that frequently accompanies cognitive impairment in post-Covid syndrome and which may compromise performance on longer test batteries.

This study therefore aimed to assess the performance on the ACE-III, representing the most comprehensive test used to assess cognition in UK memory clinics, of patients presenting to a memory clinic with cognitive impairment as part of post-Covid syndrome. In an exploratory study, a subset of individuals were also tested on the Long COVID Cognitive Assessment Battery (LCCAB), a battery of short digital tests probing attention, working memory, executive function and speed of information processing.

## 2 Methods and materials

### 2.1 *Participants*

Participants (*N* = 102) were recruited from a specialist cognitive long Covid clinic at University Hospitals Sussex NHS Trust set up specifically for this new cognitive disorder. Inclusion criteria were: (1) cognitive impairment persisting for more than three months after acute COVID-19 infection; (2) clinical features suggestive of prior acute COVID-19 infection per national guideline of symptoms. Exclusion criteria included: (1) cognitive impairment prior to COVID-19 infection; (2) co-occurrence of acute neurological disorders; (3) concurrent use of medication with potential to affect cognition; (4) pre-existing psychiatric or medical disorders with potential to affect cognition; (5) high alcohol intake; (6) recreational drug use.

### 2.2 Research design and procedure

Participants with post-covid syndrome who were considered to have significant cognitive impairment were referred by general practitioners or community-post COVID services. In addition to routine clinical assessment, self-report questionnaires assessed core features of post-Covid syndrome with potential to affect cognition, namely the Chalder Fatigue Scale (CFQ) (Chalder et al., 1993), Pittsburgh Sleep Quality Index (PSQI) (Buysse et al., 1989), generalised Anxiety Disorder 7-item Scale (GAD-7) (Spitzer et al., 2006); and Patient Health Questionnaire 9-item depression scale (PHQ-9) (Kroenke et al., 2001).

All participants completed the ACE-III. The administration of the ACE-III requires around 15–20 min and yields five individual subdomain scores (i.e., attention, memory, verbal fluency, language and visuospatial functioning) and a total maximum score of 100.

A subset of participants (*N* = 20), referred to as the ACE-III + LCCAB subgroup, additionally completed the Long COVID Cognitive Assessment Battery (LCCAB), comprised of six computer-based tasks, based on theoretical neuroscientific principles of brain-behaviour relationships (Burgess et al., 2007; Burgess and Shallice, 1996) and administered using the Gorilla platform (www.gorilla.sc). The LCCAB was administered after the ACE-III, towards the end of the assessment session. These tasks were: a simple reaction time task to assess processing speed, a modified go/no-go task to assess inhibitory control, and three tasks to assess attention to stimuli displayed on the screen (stimulus orientated thought; SOT) or attention independent of stimuli (stimulus independent thought; SIT) (See Supplementary Fig. 1) (adapted from Crum et al., 2022; Gilbert et al., 2005). Working memory was assessed using a two-condition task: the baseline condition involved judging a pair of visually presented items to identify the heaviest item, while the second condition involved judging each item's weight relative to the preceding item (adapted from Ronca et al., 2024). The intertrial interval was fixed at 5 s for the baseline condition, while in the second condition it was variable (up to 5 s), with the next trial's stimulus presented immediately following the participant's response.

Total time for the combined LCCAB tests was 15 min (SD = 2.88). Each task yielded mean reaction time and accuracy measures.

### 2.3 Statistical analysis

Statistical analyses were conducted using IBM SPSS Statistics 28.0. The normality of distribution of ACE-III performance was analysed using the Shapiro-Wilk test. Bivariate correlations between continuous variables were analysed using Spearman's rank correlation coefficient. Fatigue, sleep, anxiety and depression variables were entered into a backward elimination model of linear regression to analyse predictive value. Bonferroni correction of multiple comparisons was employed where appropriate. Cognitive performance on LCCAB tasks was classified into percentile boundaries in accordance to an age-matched normative data set, with significant impairment defined as performance at ≤5% of mean normal score. The comparison of the two groups of participants (i.e., ACE-III cohort vs ACE-III + LCCAB subgroup) to ensure representativeness was performed using the Mann-Whitney *U* test for continuous variables, and Fisher's exact test for categorical variables.

## 3 Results

### *3.1* Sociodemographic and clinical characteristics

Sociodemographic and clinical characteristics are detailed in Table 1. There was no significant difference in clinical-demographic characteristics, ACE-III cognitive performance, and fatigue and psychological variables between the ACE-III (*N* = 102) cohort and the ACE-III + LCCAB subgroup(N = 20) (Table 1). One participant from the ACE-III + LCCAB subgroup was excluded from further LCCAB analyses due to cognitive performance outside normal range in all LCCAB tasks.

**Table 1:** Sociodemographic and clinical characteristics of long COVID participants.

	ACE-III (N = 102)	ACE-III + LCCAB (N = 20)	Statistic
**Demographics**			
Age	47.60 ± 11.93	46.40 ± 13.34	*U =* 942.50, *p* = .850
*Gender*			
Females	70 (68.6%)	13 (65%)	*p* = .790
Males	32 (31.4%)	7 (35%)	
*Race*			
White	100 (98%)	20 (100%)	*p* = 1.00
Ethnic Minority	2 (2%)	0 (0%)	
Years of Education	15.69 ± 3.33	16.05 ± 4.55	*U =* 920.50, *p* = .727
*Employment Status*			
Active	87 (85.3%)	16 (80%)	
Unemployed	5 (4.9%)	1 (5%)	
Student	3 (2.9%)	0 (0%)	
Retired	7 (6.9%)	3 (15%)	

**COVID-19 History**			

Time from COVID-19 diagnosis (months)^a^	16.3 ± 6.8	17.7 ± 8.5	*U =* 837.00, *p* = .346
Positive COVID-19 biological test	64 (62.7%)	12 (60%)	*p* = .798
*Severity of COVID-19 illness*			
Non-hospitalised	85 (83.3%)	19 (95%)	*p* = .301
Hospitalised	17 (16.7%)	1 (5%)	
Ventilatory assistance	2 (2%)	0 (0%)	
**POST-COVID**			

CFQ (0–42)	32.39 ± 7.54	31.80 ± 7.30	*U =* 894.50, *p* = .595

PSQI (0–21)	10.40 ± 4.11	10.05 ± 4.48	*U =* 961.00, *p* = .954
Good Sleep Quality (≤5)	14 (13.7%)	4 (20%)	*p* = .730
Poor Sleep Quality (>5)	88 (86.3%)	16 (80%)	
GAD-7 (0–21)	10.57 ± 5.72	10.00 ± 6.02	*U =* 955.50, *p* = .923
Minimal Anxiety	23 (22.5%)	5 (25%)	*p* = 1.00
Mild Anxiety	20 (19.6%)	4 (20%)	
Moderate Anxiety	29 (28.4%)	5 (25%)	
Severe Anxiety	30 (29.4%)	6 (30%)	
PHQ-9 (0–27)	14.0 ± 6.12	13.75 ± 6.46	*U =* 960.00, *p* = .949
Minimal Depression	7 (6.9%)	2 (10%)	*p* = .945
Mild Depression	23 (22.5%)	5 (25%)	
Moderate Depression	19 (18.6%)	4 (20%)	
Moderately Severe Depression	32 (31.4%)	5 (25%)	
Severe Depression	21 (20.6%)	4 (20%)	

Table 1. Data represented as N (valid %) or Mean ± SD. Statistic result of Mann-Whitney *U* test/Fisher's exact test comparing ACE-III + LCCAB subgroup(N = 20) to ACE-III cohort (*N* = 102). CFQ: Chalder Fatigue Questionnaire; PSQI: Pittsburgh Sleep Quality Index; GAD-7: Generalised Anxiety Disorder 7-item Scale; PHQ-9: Patient Health Questionnaire 9-item scale.

a Data refers to the time from COVID-19 diagnosis to date of assessment in months.

### 3.2 Frequency of cognitive impairment on the ACE-III

**Fig. 1:**
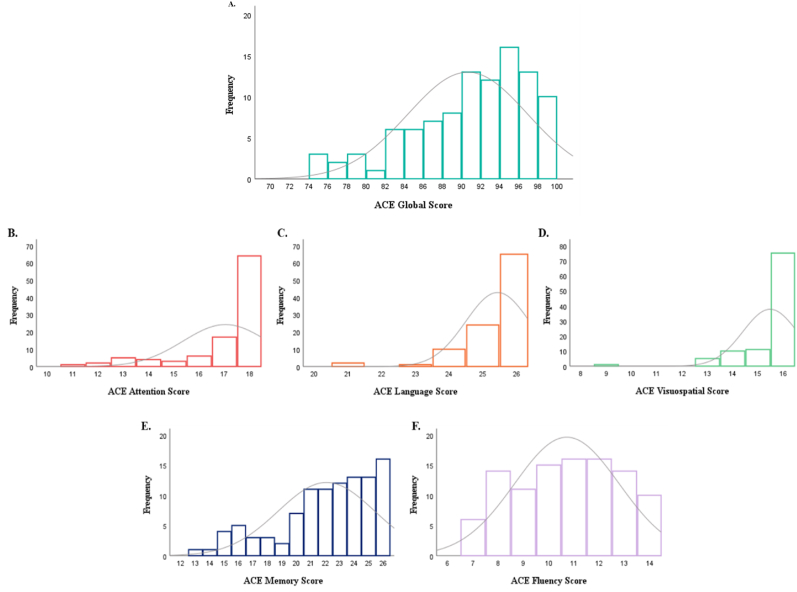
*Cognitive Performance of Long COVID Participants on the ACE-III* (N = 102). *Note.* Histograms to illustrate the distribution of cognitive performance of long COVID participants on the ACE-III (*N* = 102). (A) ACE-III Global; (B) ACE-III Attention; (C) ACE-III Language; (D) ACE-III Visuospatial; (C) ACE-III Memory; (F) ACE-III Fluency. Horizontal and longitudinal axes indicate cognitive score and frequency count for each score, respectively.

The cognitive performance of long COVID participants on the ACE-III are detailed in Table 2 and performance distributions are illustrated in Fig. 1. A score of ≤84 has been considered to have a sensitivity of 0.92 and specificity of 0.63 for differentiating mild cognitive impairment from normal cognition (Potts et al., 2021). Accordingly, using ≤84 as a score indicating cognitive impairment, 15.7% (*N* = 16) of participants were impaired and 84.3% (*N* = 86) performing in the normal range(M = 91, SD = 6). The maximum score was attained by 62.7% (N = 64) in attention, 63.7% (N = 65) in language, and 73.5% (N = 75) in visuospatial subdomains.

**Table 2:** Cognitive Performance of Long COVID Participants on the ACE-III (N = 102).

ACE-III Score	ACE-III (*N* = 102)	ACE-III + LCCAB (N = 19)	Statistic
Global (0–100)	90.65 ± 6.27	90.95 ± 5.19	*U =* 942.50, *p* = .850
Attention (0–18)	17.04 ± 1.68	17.05 ± 1.23	*U =* 792.50, *p* = .157
Memory (0–26)	22.08 ± 3.36	22.15 ± 3.62	*U =* 892.50, *p* = .583
Fluency (0–14)	10.72 ± 2.08	10.90 ± 1.83	*U =* 873.00, *p* = .490
Language (0–26)	25.44 ± 0.95	25.55 ± 0.51	*U =* 920.00, *p* = .685
Visuospatial (0–16)	15.48 ± 1.08	15.68 ± 0.82	*U =* 870.00, *p* = .351

Table 2. Data represented as N (valid %) or Mean ± SD. MCI: Mild cognitive impairment. Statistic result of Mann-Whitney *U* test/Fisher's exact test comparing ACE-III + LCCAB subgroup(N = 19) to ACE-III cohort (*N* = 102).

### 3.3 Frequency of cognitive impairment on the LCCAB

**Fig. 2:**
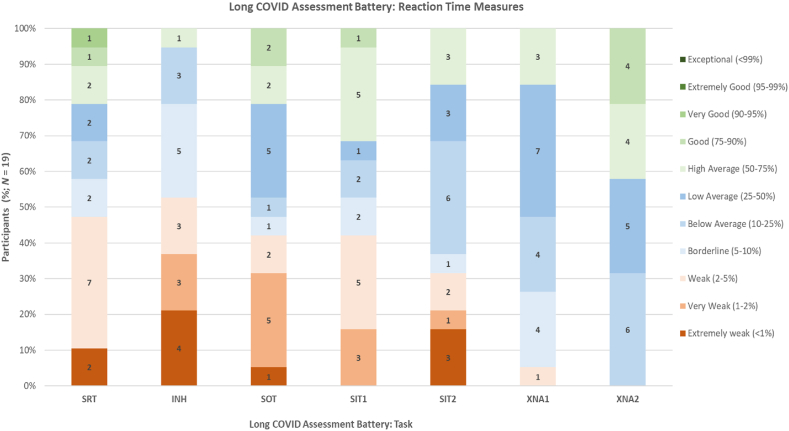
*Cognitive Performance of Long COVID Participants on the LCCAB* (N = 19). *Note.* Cognitive performance (mean reaction time) of long COVID participants on the LCCAB (*N* = 19). SRT: Simple Reaction Time; SOT: Stimulus Oriented Thought; SIT1: Stimulus Independent Thought 1; SIT2: Stimulus Independent Thought 2; XNA1: Working Memory Baseline Task; XNA2: Working Memory Task.

The cognitive performance of long COVID participants (*N* = 19) on the LCCAB is illustrated in Fig. 2. There was no significant difference in ACE-III cognitive performance between the ACE-III cohort (*N* = 102) and ACE-III + LCCAB subgroup(N = 19) (Table 2), indicating that on this measure this subset was representative of the larger study cohort.

Participants exhibited significant impairment (defined as a performance at or below the 5th percentile) in reaction time measures for processing speed (47.4%, *N* = 9), inhibitory control (52.6%, N = 10), attention (**SOT**: 42.1%, N = 8; **SIT1**: 42.1%, N = 8; **SIT2**: 31.6%, N = 8), and working memory baseline (**XNA1**: 5.3%, *N* = 1) (Fig. 2). A lower proportion of participants displayed impairment in error scores for inhibitory control (5.3%, *N* = 1), attention (**SOT**: 5.3%, N = 1; **SIT1**: 10.5%, N = 2; **SIT2**: 15.8%, N = 3), and working memory baseline (15.8%, *N* = 3). However, the XNA2 error score, indicative of working memory performance, revealed impairment in 41.1% (*N* = 8) of participants.

Overall, 89.47% (*N* = 17) of long COVID participants assessed using these digital tests revealed significant impairment on at least one cognitive task, with only 10.53% (*N* = 2) without objective cognitive impairment.

### 3.4 Contributors to ACE-III and LCCAB cognitive performance

Anxiety, depression, fatigue and sleep scores did not significantly correlate with ACE-III or LCCAB cognitive performance. However, sleep scores did significantly correlate with LCCAB attention tasks; specifically, a strong positive correlation with SOT (*r* = 0.63, *p* < .01), and a moderate positive correlation with SIT1 (*r* = 0.49, *p* < .05) and SIT2 (*r* = 0.50, *p* < .05). This correlation did not yield significance following a conservative correction for multiple comparisons. Backwards stepwise linear regression models were conducted with psychological and fatigue variables as predictors for either ACE-III or LCCAB cognitive scores. Fatigue and anxiety scores emerged as marginally significant predictors of ACE-III Global (*R*^*2*^ (adjusted) = 0.021, *F*_(1, 100)_ = 3.200, *p* = .077) and ACE-III Fluency scores (*R*^*2*^ (adjusted) = 0.026, *F*_(1, 100)_ = 3.735, *p* = .056), respectively. Sleep scores significantly predicted SOT (*R*^*2*^ (adjusted) = 0.221, *F*_(1, 17)_ = 6.099, *p* = .024) and SIT2 *R*^*2*^ (adjusted) = 0.223, *F*_(3, 15)_ = 6.177, *p* = .024), while sleep, fatigue and depression scores significantly predicted SIT1 (*R*^*2*^ (adjusted) = 0.348, *F*_(3, 15)_ = 4.203, *p* = .024).

## 4 Discussion

The aim of this study was to assess performance on the ACE-III cognitive battery of people with post-Covid syndrome self-reporting cognitive difficulties. The mean ACE-III score from 102 participants was 91/100 (SD 6) with 15.7% (16/102) scoring at or below the level considered to represent objective cognitive impairment. In contrast, of the n = 20 individuals who also completed the LCCAB, a battery of tests assessing processing speed, inhibitory control, attention, and working memory, 89.47% were significantly impaired on at least one cognitive task, with attention, executive function and processing speed being the cognitive domains most affected.

The high average score on ACE-III and low percentage of those found to fall below the ACE-III score considered indicative of cognitive impairment is likely to be due to the fact that the cognitive domains affected in post-Covid syndrome are not adequately captured in this test, which was designed to address those domains most affected in diseases causing dementia, such as Alzheimer's disease. In these dementia disorders episodic memory, language and visuospatial function are commonly affected, and this is reflected in these domains being measured using brief pen and paper assessments such as the ACE-III and MMSE. This observation is reinforced by the findings from a sub-study using the LCCAB which was designed specifically to address the pattern of cognitive impairment associated with post-Covid syndrome, and on which the overwhelming majority, nearly 90% of those tested, exhibited significant impairment. Specifically, the widespread impairment in reaction time for processing speed (47.4%), inhibitory control (52.6%), and attention tasks (31.6–42.1%) correspond to reports of ‘brain fog’, slower processing and disorientation, while observed working memory impairments (41.1%) may reflect the difficulties in multitasking and forgetfulness described by patients (Callan et al., 2022). These objective LCCAB findings not only provide quantifiable evidence for the subjective cognitive complaints commonly reported in post-COVID syndrome, but also suggest that computerised cognitive batteries may be more sensitive in capturing the specific cognitive profile associated with this condition.

It is noteworthy, however, that not all participants displayed objective cognitive deficits in LCCAB performance, despite all presenting with subjective cognitive complaints. This might mean that the short LCCAB does not adequately capture the full spectrum of cognitive impairment in individuals with post-covid syndrome. It is possible that a more comprehensive cognitive assessment of the type administered by trained neuropsychologists may identify a greater range of objective cognitive deficits but such tests are resource- and time-intensive and cannot be undertaken within a routine clinic appointment. However, an alternative explanation is that there is a mismatch between subjective cognitive complaints and objective cognitive performance in this patient cohort, an observation arising from previous work (Schild et al., 2023; Zhao et al., 2022).

Our analysis revealed significant associations between LCCAB performance, particularly on attention tasks, and sleep quality, while ACE-III scores showed minimal correlations with other post-COVID symptoms. Sleep scores significantly predicted performance on several LCCAB attention measures, with a combination of sleep, fatigue, and depression scores predicting SIT1 performance, suggesting that cognitive impairment in post-covid syndrome may be partially modulated by other symptoms. This aligns with established sleep-attention associations (Kirszenblat and van Swinderen, 2015) and corroborates recent COVID-19 research (Delgado-Alonso et al., 2022). However, as these factors do not account for all variance in cognitive performance, direct effects of SARS-CoV-2 on cognition may exist independently. These findings highlight the limitations of ACE-III and the advantages of LCCAB in capturing the full spectrum of cognitive changes in post-COVID syndrome, guiding future research and potential interventions.

In conclusion, a legacy cognitive test such as the ACE-III, designed for clinic assessment of Alzheimer's disease and other dementia disorders, poorly captures the impairment associated with post-Covid syndrome, with its very different profile of affected domains. Considerable evidence demonstrates that cognitive tests used in research, such as the LCCAB in this study, are more effective at detecting post-COVID cognitive impairment than standard clinical assessments. However, before these tools can be widely adopted in clinical practice, they require further validation studies and comprehensive evaluation of their psychometric properties. The emergence of new cognitive disorders such as post-covid syndrome highlights the urgent need for updated cognitive test batteries in routine clinical practice.

## CRediT authorship contribution statement

**Aysha Mohamed Rafik Patel:** Writing – review & editing, Writing – original draft, Methodology, Investigation, Formal analysis. **Gina Gilpin:** Writing – review & editing. **Anna Koniotes:** Writing – review & editing, Methodology, Data curation. **Catherine Warren:** Writing – review & editing, Methodology, Data curation. **Cian Xu:** Writing – review & editing. **Paul W. Burgess:** Writing – review & editing, Methodology. **Dennis Chan:** Writing – review & editing, Writing – original draft, Supervision, Methodology.

## Funding

Dennis Chan is funded by the National Institute of Health Research, the Wellcome Trust, Alzheimer's Research UK and the UK Dementia Research Institute.

## Declaration of competing interest

The authors declare that they have no known competing financial interests or personal relationships that could have appeared to influence the work reported in this paper.

## Data Availability

Data will be made available on request.
