# Supplementary material for: Clinic evaluation of cognitive impairment in post-COVID syndrome: Performance on legacy pen-and-paper and new digital cognitive tests
Source: Brain Behav Immun Health. 2024 Dec 1;43:100917. doi: 10.1016/j.bbih.2024.100917 (PMC11665294; doi:10.1016/j.bbih.2024.100917)
Supplement: Multimedia component 1 [file mmc1.pdf]

Supplementary Figure 1.

The Long COVID Cognitive Assessment Battery (LCCAB) – Measures of Attention

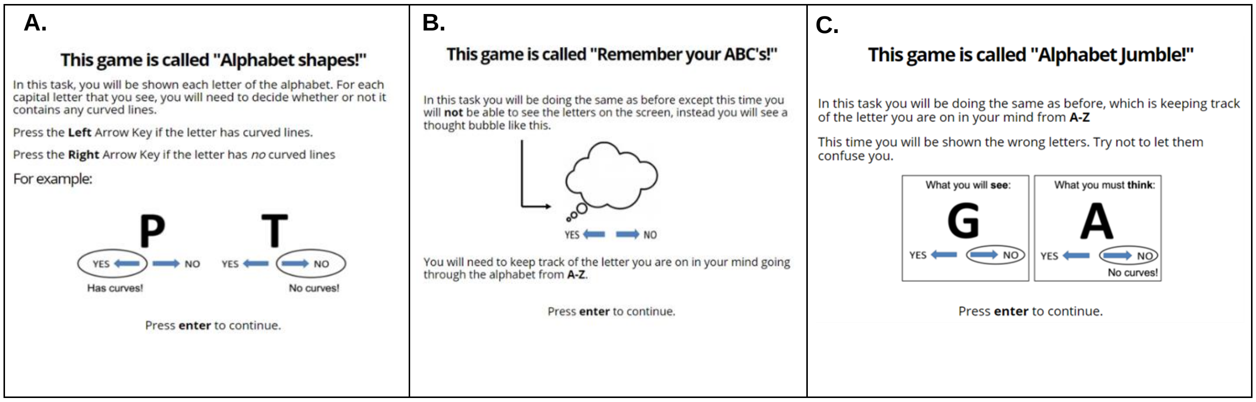

Figure 1. (A) SOT: Stimulus Oriented Thought. Participants classified visually presented alphabet letters based on the presence of curved lines (26 trials). (B) SIT1: Stimulus Independent Thought 1. Participants classified internally generated alphabet letters based on the presence of curved lines (26 trials). Letters were not visually presented and thought bubbles were displayed. (C) SIT2: Stimulus Independent Thought 2. Participants classified internally generated alphabet letters based on the presence of curved lines. Distractor letters were displayed (26 trials). Stimulus duration in all tasks was self-paced with a maximum trial length of 15 seconds.
